#  Cobertura do Programa Bolsa Família e fatores associados à realização de procedimentos odontológicos no Brasil, entre 2007 e 2011: um estudo ecológico

**DOI:** 10.1590/0102-311XPT200622

**Published:** 2023-07-17

**Authors:** Beatriz Carriconde Colvara, Irene Fanny Ritzel, Violeta Rodrigues Aguiar, Juliana Balbinot Hilgert, Roger Keller Celeste

**Affiliations:** 1 Universidade Federal do Rio Grande do Sul, Porto Alegre, Brasil.

**Keywords:** Serviços de Saúde Bucal, Uso de Serviços de Saúde, Pobreza, Disparidades Socioeconômicas em Saúde, Dental Health Services, Health Services, Poverty, Socioeconomic Disparities in Health, Servicios de Salud Dental, Uso de Servicios de Salud, Pobreza, Disparidades Socieonómicas en Salud

## Abstract

No Brasil, houve expansão da cobertura de serviços odontológicos na atenção primária à saúde (APS), e a ênfase do trabalho dos profissionais mudou para incluir mais esforços na prevenção e no diagnóstico. Entretanto, pouco se sabe sobre a influência da cobertura do Programa Bolsa Família no uso desses serviços. Esta pesquisa avaliou a associação entre cobertura municipal do Programa Bolsa Família e uso de serviços odontológicos. Este estudo ecológico, realizado com dados dos 5.570 municípios brasileiros, estimou, por meio de regressões logísticas, o impacto da variação de cobertura do Programa Bolsa Família, das Estratégias Saúde da Família (ESF) e das equipes de saúde bucal (EqSB) no número de procedimentos odontológicos restauradores, coletivos, preventivos e exodontias realizados via Sistema Único de Saúde (SUS) entre os períodos 2007/2008 e 2010/2011. Os percentuais de municípios em que houve aumento das taxas de procedimentos preventivos, coletivos, restauradores e exodontias foram de 46%, 59,8%, 52,5% e 44,2%, respectivamente. No modelo ajustado, em municípios com maior cobertura do Bolsa Família houve menos chances de aumentar a ocorrência de procedimentos coletivos (OR = 0,91; IC95%: 0,79-1,04) e preventivos (OR = 0,92; IC95%: 0,80-1,05) e mais chances de elevar as taxas de procedimentos restauradores (OR = 1,11; IC95%: 0,97-1,28) e exodontias (OR = 1,10; IC95%: 0,95-1,27). A expansão na taxa de cobertura das EqSB esteve associada significativamente a uma chance maior de aumento do número de procedimentos preventivos, restauradores e exodontias. Conclui-se que a cobertura das EqSB foi a principal variável associada à ampliação da quantidade de procedimentos odontológicos realizados no serviço público.

## Abstract (en)

In Brazil, there has been an expansion of the coverage of dental services in primary health care (PHC), and the focus of the services has changed to include more efforts in prevention and diagnosis. However, little is known about the influence of the coverage of the Brazilian Income Transfer program on the use of dental services. Our study evaluates the association between municipal coverage of the Brazilian Income Transfer Program and the use of dental services. This ecological study conducted with data from the 5,570 Brazilian municipalities estimates, via logistic regressions, the impact of the coverage of the Brazilian Income Transfer program, the Family Health Strategies (FHS), and the oral health teams on the number of restoration, colective, prevention, and dental extraction procedures performed by the Brazilian Unified National Health System (SUS) between the periods 2007/2008 and 2010/2011. The percentage of municipalities that increased the rates of prevention, colective, restoration, and extraction procedures was 46%, 59.8%, 52.5%, and 44.2%, respectively. In the adjusted model, municipalities with increased coverage of Brazilian Income Transfer program were less likely to increase colective (OR = 0.91; 95%CI: 0.79-1.04) and prevention (OR = 0.92; 95%CI: 0.80-1.05) procedures, and they were more likely to increase rates of restoration (OR = 1.11; 95%CI: 0.97-1.28) and dental extraction (OR = 1,10; 95%CI: 0.95-1.27) procedures. The increase in the coverage rate of the oral health teams was significantly associated with a higher chance of an increase in prevention, restoration, and extraction procedures. We conclude that the coverage of oral health teams was the main variable associated with the increase in dental procedures performed in the public service.

## Resumen

En Brasil, hubo una expansión de la cobertura de servicios odontológicos en la atención primaria a la salud, y el énfasis del trabajo de los profesionales cambió para incluir más esfuerzos en la prevención y en el diagnóstico. Sin embargo, poco se sabe sobre la influencia de la cobertura del Programa Bolsa Familia en el uso de los servicios odontológicos. El presente estudio evaluó la asociación entre la cobertura municipal del Programa Bolsa Familia y uso de servicios odontológicos. Un estudio ecológico realizado con datos de los 5.570 municipios brasileños estimó, a través de regresiones logísticas; el impacto de la variación de cobertura del Programa Bolsa Familia, de las Estrategias de Salud de la Familia (ESF) y de los equipos de salud bucal (EqSB) en el número de procedimientos odontológicos restauradores, colectivos, preventivos y exodoncias realizados a través del Sistema Único de Salud (SUS) entre los períodos 2007/2008 y 2010/2011. El porcentaje de municipios que aumentaron las tasas de procedimientos preventivos, colectivos, restauradores y exodoncias fue del 46 %, 59,8 %, 52,5 % y 44,2 %, respectivamente. En el modelo ajustado, los municipios con aumento de la cobertura de Programa Bolsa Familia tuvieron menos probabilidades de aumentar procedimientos colectivos (OR = 0,91; IC95%: 0,79-1,04), y preventivos (OR = 0,92; IC95%: 0,80-1,05), y más probabilidades de aumentar las tasas de procedimientos restauradores (OR = 1,11; IC95%: 0,97-1,28) y exodoncias (OR = 1,10; IC95%: 0,95-1,27). El aumento en la tasa de cobertura de EqSB se asoció significativamente con una mayor probabilidad de aumento en procedimientos preventivos, restauradores y exodoncias. Se concluye que la cobertura de las EqSB fue la principal variable asociada al aumento de los procedimientos odontológicos realizados en el servicio público.

## Introdução

Programas de transferência de renda objetivam aliviar os efeitos da pobreza a curto prazo e, a longo prazo, aspiram quebrar o ciclo intergeracional da pobreza. Centram-se, prioritariamente, nas crianças, visando que se tornem adultos produtivos e autossuficientes ^1^. Esses programas podem ser divididos em diretos ou indiretos: o primeiro consiste em transferência de dinheiro para os beneficiários, enquanto o segundo se refere a transferências nas formas de produtos ou serviços (alimentos, gás de cozinha, material escolar, consultas médicas e/ou odontológicas etc.). Outra tipologia dos programas de transferência de renda diz respeito à exigência ou não de condicionalidades, as quais precisam ser cumpridas para que o benefício continue sendo distribuído ao longo do tempo. Essas condicionalidades podem incluir frequência escolar, esquema vacinal completo, monitoramento de crescimento infantil pelas equipes de saúde ^2^ ou, ainda, participação em outros programas governamentais ^3^. Tais determinações também são uma forma de incentivar as famílias a cumprirem com alguns compromissos relativos à própria saúde dos beneficiários.

O Bolsa Família, que em 2021 foi substituído por um novo programa de transferência de renda chamado Auxílio Brasil, foi um dos maiores programas de transferência de renda a operar mundialmente. Ele foi criado no ano de 2004 ^4^ e beneficiou mais de 18 milhões de famílias em todo o território nacional, segundo dados oficiais ^5^. O benefício médio, em outubro de 2021, era de BRL 87,50 (USD 17,10), transferido preferencialmente para a mulher chefe da família, que poderia utilizá-lo da forma que desejasse. Esse programa esteve associado com aumento das chances de acompanhamento do crescimento infantil e de vacinação em menores de sete anos ^6^. A taxa de mortalidade infantil resultante de causas gerais e de fatores relacionados à pobreza diminuiu à medida que a cobertura do Programa Bolsa Família aumentou. Uma das explicações possíveis para esse evento é a influência das condicionalidades relativas à saúde infantil ^7^. Além disso, estudos concluem que programas de transferência condicional de renda são capazes de aumentar o uso de serviços preventivos ^8^^,^^9^, além de incentivarem a adoção de comportamentos saudáveis por parte dos beneficiários ^10^. De forma semelhante, esses programas poderiam influenciar o uso do serviço odontológico, mesmo que tal atendimento não faça parte das condicionalidades exigidas por alguns programas, como no caso do Bolsa Família. Evidência recente sobre o efeito de programas de transferência de renda é inconclusiva, no entanto, indica que o uso de serviços odontológico parece não aumentar ^11^^,^^12^^,^^13^^,^^14^^,^^15^.

No Brasil, houve expansão da cobertura de serviços odontológicos na atenção primária à saúde (APS) ^16^^,^^17^, concomitantemente ao relato de redução, ainda que lenta, da desigualdade no uso de serviços ^18^^,^^19^. A utilização dos serviços depende de uma multiplicidade de fatores, como necessidade de saúde, elementos contextuais e aspectos intrínsecos ao serviço de saúde ^20^. Na odontologia, a ênfase do trabalho dos profissionais mudou para incluir mais esforços na prevenção e no diagnóstico, e menos em extrações dentárias ^21^. Os principais preditores para consulta de rotina odontológica em crianças são o nível econômico, a escolaridade materna e a mãe ter recebido orientações sobre prevenção ^22^. Assim, é plausível que o aumento da cobertura do Programa Bolsa Família possa modificar o uso do serviço de saúde odontológico, uma vez que há condicionalidades para o uso de serviços de saúde e aumento da renda das famílias. Este estudo objetiva avaliar a associação entre a cobertura municipal do programa brasileiro de transferência de renda direta e condicionada, Bolsa Família, e o uso de serviços odontológicos no sistema de saúde público de acesso universal em todos os municípios brasileiros.

## Métodos

### Desenho do estudo

Este é um estudo ecológico em nível municipal de agregação que investiga mudanças no tempo. O período inicial é a média dos anos 2007 e 2008 e o período final é a média de 2010 e 2011. Esses dois períodos foram selecionados pela disponibilidade dos dados cruzados para as variáveis de cobertura do Programa Bolsa Família e uso dos serviços odontológicos, bem como pelas informações obtidas do censo de 2010 para as demais covariadas. Foram coletados e analisados os dados para todos os 5.570 municípios brasileiros. A descrição do estudo segue os critérios indicados pelo *REporting of studies Conducted using Observational Routinely-collected Data* (RECORD) ^23^. 

### Variáveis e fontes de dados

Procedimentos odontológicos realizados no Sistema Único de Saúde (SUS) foram as variáveis dependentes deste estudo. Tais informações foram coletadas do Sistema de Informações Ambulatoriais do Sistema Único de Saúde (SIA-SUS), por meio do Tabnet, e divididas em quatro grupos, a saber: procedimentos coletivos, restauradores, preventivos e exodontias, conforme elencados por Celeste et al. ^21^. Em janeiro de 2008, foram feitas alterações na tabela de códigos e na nomenclatura de alguns procedimentos realizados. Para manter a comparabilidade, foram extraídos os dados correspondentes aos códigos e procedimentos elencados inicialmente.

A cobertura do Programa Bolsa Família foi a principal variável explicativa (independente) de interesse. Ela foi construída da seguinte forma: percentual de famílias atendidas pelo programa em cada município em relação à estimativa de famílias pobres que se enquadram no perfil de inclusão do programa. Em 2007 e 2008, o indicador foi calculado utilizando como denominador a variável “estimativa de famílias pobres - PNAD 2006” (famílias com renda familiar *per capita* de até BRL 140,00). A partir de 2010, o indicador foi calculado adotando como denominador a variável “estimativa de famílias pobres - perfil Bolsa Família - censo 2010”. Os dados foram obtidos do Departamento de Informática do Sistema Único de Saúde (DATASUS), por meio do aplicativo Tabnet (http://www2.datasus.gov.br/tabnetmobile/page_about2.html).

A cobertura populacional das Estratégias Saúde da Família (ESF) e das equipes de saúde bucal (EqSB) foram consideradas variáveis independentes de confusão. Elas são percebidas como indicadores de acesso ao serviço odontológico (preditor do uso do serviço, possibilita a análise da situação dos serviços ofertados naquele período). Como numerador, foi extraído o número de equipes por município do Sistema de Cadastro Nacional de Estabelecimentos de Saúde (CNES) e como denominador utilizou-se a população municipal estimada pelo Instituto Brasileiro de Geografia e Estatística (IBGE). Com isso, calculou-se a taxa de equipes por 100 mil habitantes por ano em cada município.

As demais variáveis independentes (covariadas) foram: (a) renda *per capita* média por habitante em nível municipal; (b) população de mulheres/população residente feminina por município; (c) população idosa por município; (d) população de crianças de até 14 anos por município; (e) porcentagem da população em situação de extrema pobreza. Com exceção da covariada “população de crianças de até 14 anos por município”, que foi extraída de dados do IBGE, todas as demais provêm do Atlas de Desenvolvimento Humano no Brasil ^24^.

### Análise estatística

As taxas anuais para cada grupo de procedimento odontológico usado como variável dependente foram calculadas por 100 habitantes/ano, enquanto as taxas de cobertura das ESF e das EqSB foram calculadas para cada 10 mil habitantes/ano. Posteriormente, as variáveis foram categorizadas de forma a identificar municípios que aumentaram as taxas das quatro variáveis dependentes entre os dois períodos e os que aumentaram, mantiveram ou reduziram as taxas de cobertura de ESF e EqSB. A variável de cobertura do Bolsa Família na linha de base (2007/2008) foi categorizada em menor que 33 pontos percentuais (p.p.), entre 33 e 66p.p. e maior que 66p.p. A mudança na cobertura do programa foi categorizada em quatro aspectos: municípios que aumentaram mais de 40p.p.; que aumentaram entre 20 e 40p.p.; que aumentaram até 20p.p.; ou que reduziram a cobertura entre os períodos analisados. A categorização das demais covariadas aconteceu a partir da mediana, resultando em dois grupos de mesmo tamanho.

A análise bivariada foi realizada por meio do teste qui-quadrado e a análise por modelos de regressão logística foi feita utilizando como desfechos os quatro grupos de procedimentos. A modelagem *stepwise backward* foi executada inicialmente com o modelo cheio (todas as variáveis) e considerando p > 0.25 para remoção. A variável de cobertura do Programa Bolsa Família foi mantida fixa em todos os modelos testados, independentemente do valor de p. A qualidade dos ajustes foi medida usando o critério de informação de Akaike (AIC). A edição dos dados e a análise estatística foram realizadas no software livre R, versão 4.1.3 (http://www.r-project.org).

## Resultados

Entre os períodos de 2007/2008 e 2010/2011, os percentuais de municípios em que houve aumento das taxas de procedimentos coletivos, preventivos, restauradores e exodontias foram de 59,8%, 46,0%, 52,5% e 44,2%, respectivamente (Tabela 1). Dos 5.570 municípios brasileiros, 4.558 tiveram algum aumento na taxa de cobertura do Programa Bolsa Família, o que equivale a quase 82% dos municípios. Da mesma forma, houve aumento da cobertura de ESF e EqSB em 55,3% e 49,1%, respectivamente. Ademais, o aumento da cobertura de ESF parece estar associado às variações dos procedimentos restauradores no país (< 0,01).

**Tabela 1:** Frequência de municípios brasileiros que aumentaram as taxas de procedimentos odontológicos coletivos, preventivos, restauradores e exodônticos entre os períodos de 2007/2008 e 2010/2011.

	Total		Aumento de procedimentos coletivos		Aumento de procedimentos preventivos		Aumento de procedimentos restauradores		Aumento de procedimentos exodônticos	
	n	%	%	Valor de p	%	Valor de p	%	Valor de p	%	Valor de p
Total	5.570	100,0	59,8	-	46,0	-	52,5		44,2	-
Porte populacional										
Maior população absoluta	2.831	100,0	58,0	< 0,01	45,8	0,79	51,0	0,03	57,6	< 0,01
Menor população absoluta (até 10.747 habitantes)	2.734	100,0	61,7		46,2		54,0		30,4	
População idosa residente										
Maior população de idosos	2.831	100,0	58,2	0,02	46,0	1,00	51,9	0,37	57,8	< 0,01
Menor população de idosos (até 2.220 idosos residentes)	2.734	100,0	61,4		46,0		53,1		30,2	
População feminina residente										
Maior população feminina	2.998	100,0	59,6	0,77	45,2	0,20	51,4	0,09	49,2	< 0,01
Menor população feminina (até 5.304 mulheres residentes)	2.567	100,0	60,0		47,0		53,7		38,4	
População de crianças de até 14 anos residentes										
Maior população de crianças	2.782	100,0	63,0	< 0,01	48,6	< 0,01	55,8	< 0,01	48,0	< 0,01
Menor população de crianças (até 2.765 crianças residentes)	2.783	100,0	56,6		43,5		49,2		40,5	
Porcentagem da população em extrema pobreza										
Maior % da população em extrema pobreza	2.796	100,0	62,9	< 0,01	49,0	< 0,01	55,7	< 0,01	45,3	0,11
Menor % da população em extrema pobreza (até 6% da população)	2.769	100,0	56,7		43,1		49,2		43,2	
Renda *per capita* média										
Maior renda *per capita* média	2.830	100,0	56,7	< 0,01	43,1	< 0,01	49,1	< 0,01	43,7	0,47
Menor renda *per capita* média (até BRL 465,00)	2.734	100,0	63,1		49,0		56,0		44,7	
Cobertura das ESF										
Aumentou	3.078	100,0	59,4	0,28	46,9	0,31	52,6	0,65	46,0	< 0,01
Manteve	189	100,0	55,6		47,1		55,6		35,4	
Reduziu	2.298	100,0	60,7		44,8		52,1		42,6	
Cobertura das EqSB										
Aumentou	2.733	100,0	60,4	0,23	49,2	< 0,01	57,0	< 0,01	51,7	< 0,01
Manteve	1.861	100,0	60,1		44,4		50,8		35,4	
Reduziu	971	100,0	57,4		40,3		43,2		40,2	
Mudança de cobertura do Programa Bolsa Família entre 2007/2008 e 2010/2011										
Aumentou > 40p.p.	10.61	100,0	60,3	0,32	50,7	< 0,01	59,7	< 0,01	48,4	0,01
Aumentou 20-40p.p.	1.416	100,0	59,5		45,5		52,4		43,7	
Aumentou até 20p.p.	2.081	100,0	58,6		43,3		50,1		43,7	
Reduziu	1.007	100,0	62,1		47,6		50,0		41,6	
Cobertura do Programa Bolsa Família na linha de base (2007-2008)										
< 33%	886	100,0	56,8	0,06	45,0	0,41	55,1	0,14	51,7	< 0,01
De 33%-66%	2.365	100,0	59,7		47,1		52,8		45,7	
> 66%	2.314	100,0	61,2		45,4		51,2		39,9	

EqSB: equipes de saúde bucal; ESF: Estratégia Saúde da Família; p.p.: pontos percentuais.

Com relação à cobertura do Programa Bolsa Família, as análises bivariadas e por regressão não encontraram associação significativa entre a cobertura do programa e os procedimentos odontológicos. Na análise bivariada, a taxa de cobertura das EqSB esteve associada com o aumento nas taxas de três das quatro categorias de procedimentos odontológicos: preventivos, restauradores e exodontias. De forma semelhante, na análise final por regressão ajustada, foi possível identificar que um aumento na taxa de cobertura das EqSB esteve significativamente associado a uma maior chance de as taxas de procedimentos preventivos (OR = 1,41; IC95%: 1,21-1,64), restauradores (OR = 1,76; IC95%: 1,51-2,05) e exodontias (OR = 1,41; IC95%: 1,20-1,64) elevarem no município, com exceção dos procedimentos coletivos (OR = 1,14; IC95%: 0,98-1,33), em comparação com aqueles que reduziram as taxas de cobertura das EqSB (Tabela 2).

**Tabela 2:** Modelos finais analisando o aumento na taxa de quatro categorias de procedimentos odontológicos e a taxa de cobertura do Programa Bolsa Família, das Estratégias Saúde da Família (ESF) e das equipes de saúde bucal (EqSB) entre os períodos de 2007/2008 e 2010/2011 nos 5.570 municípios brasileiros.

	Aumento de procedimentos coletivos *		Aumento de procedimentos preventivos **		Aumento de procedimentos restauradores ***		Aumento de procedimentos exodônticos ^#^	
	OR	IC95%	OR	IC95%	OR	IC95%	OR	IC95%
Cobertura do Programa Bolsa Família								
Aumentou > 40p.p.	1,21	0,99-1,47	1,49	1,23-1,80				
Aumentou até 20-40p.p.	1,12	0,95-1,31	1,12	0,95-1,31				
Aumentou até 20p.p.	1,00		1,00		1,00		1,00	
Reduziu	1,12	0,95-1,30	1,18	1,02-1,38	1,01	0,89-1,17	0,98	0,83-1,15
Cobertura das EqSB								
Aumentou	1,14	0,98-1,33	1,41	1,20-1,61	1,75	1,51-2,04	1,41	1,20-1,63
Manteve	1,09	0,93-1,28	1,14	0,97-1,34	1,28	1,09-1,51	1,03	0,87-1,22
Reduziu	1,00		1,00		1,00		1,00	
Cobertura das ESF								
Aumentou			1,05	0,94-1,17	0,97	0,86-1,08		
Manteve			1,31	0,96-1,80	1,40	1,02-1,91		
Reduziu			1,00		1,00			

p.p.: pontos percentuais.

Modelos finais ajustados para:

* Cobertura inicial do Bolsa Família, porcentagem de mulheres, idosos e crianças de até 14 anos;

** Cobertura inicial do Bolsa Família, porcentagem da população em extrema pobreza e renda *per capita* média;

*** Porte populacional do município, porcentagem de crianças de até 14 anos e renda *per capita* média;

^#^ Cobertura inicial do Bolsa Família, porte populacional do município, porcentagem de mulheres, crianças de até 14 anos e idosos.

## Discussão

A expansão da cobertura das EqSB esteve associada ao aumento de ocorrência dos procedimentos preventivos, restauradores e exodontias entre os períodos 2007/2008 e 2010/2011. Os procedimentos coletivos foram exceção, os quais já têm altas taxas, sendo a categoria de procedimentos que mais aumentou entre os períodos avaliados. Ao longo desse tempo, 82% dos municípios aumentaram as taxas de cobertura do Programa Bolsa Família, assim como 55% e 49% expandiram as taxas de cobertura das ESF e das EqSB, respectivamente. Diferentemente da hipótese inicialmente levantada, não foram identificadas associações estatisticamente significantes entre a ampliação da cobertura do Programa Bolsa Família e o aumento nas taxas das quatro categorias de procedimentos analisadas.

A variação da cobertura do Programa Bolsa Família não impactou no uso dos serviços odontológicos de forma consistente em todos os procedimentos. A disponibilidade conjunta de um serviço de APS e de um programa de transferência de renda parece ter uma eficácia adicional quando comparada com a oferta unicamente de um programa de transferência de renda ^25^, porém a qualidade dos serviços é peça importante dessa equação. No Brasil, apenas 25% das EqSB são consideradas boas quanto à qualidade da assistência à saúde bucal ^26^. Para além da qualidade e disponibilidade dos serviços ofertados, dependendo do valor transferido, diferentes resultados poderão ser esperados. Aportes pequenos de renda podem não ser capazes de alterar situações estruturais, no entanto, não é do nosso conhecimento estudo que tenha testado o recebimento de diferentes valores de transferência de renda. A possibilidade de efeitos de limiares, abaixo dos quais não há mudança, é plausível tanto para o valor do benefício como para a cobertura do programa, visto que uma ampla cobertura poderia ter maior capacidade de modificar contextos. Desconhecemos, também, programas que incluam condicionalidades relativas à saúde bucal, mas é possível que sua inclusão possa modificar o uso desses serviços. Por fim, é possível que barreiras estruturais de vida dificultem ou impeçam o acesso e uso dos serviços ^13^, como questões relativas e trabalhos eventuais em conflito com horário dos serviços de saúde, falta de transporte público, entre outras situações.

Em cenários de piores condições socioeconômicas se concentra o maior número de jovens usuários do serviço público odontológico ^27^. Nos contextos de baixa renda também identifica-se maior chance de câncer oral, maior prevalência de cárie e perda dentária, além de baixa qualidade de vida associada à saúde bucal ^28^. Nesse sentido, o aumento da cobertura do Programa Bolsa Família e a focalização adequada nos grupos mais vulneráveis são bastante importantes e desejáveis, mas também deve ser considerado um valor de transferência adequado, que atinja a renda mínima necessária para uma vida saudável, custeando determinantes da saúde, como alimentação e moradia ^29^.

Nossos achados mostram que a variação da cobertura das EqSB impactou o uso de procedimentos preventivos, restauradores e exodontias via SUS, confirmando estudos prévios ^13^^,^^16^^,^^21^. A expansão da oferta de serviços tende a aumentar o uso, especialmente em países como o Brasil, onde há uma grande demanda reprimida. Segundo Corrêa & Celeste ^16^, a incorporação de EqSB às ESF parece ser a estratégia mais efetiva para o aumento de indicadores de utilização do serviço odontológico. No ano de 2004, a implementação da *Política Nacional de Saúde Bucal*^30^ foi um marco importante para ampliar o acesso público ao dentista e gerou significativa redução no percentual de indivíduos que nunca compareceram a consultas odontológicas entre os anos 1998 e 2013 ^18^. Entre 1999 e 2011, a produção odontológica aumentou quase 50% ^16^. Com relação aos tipos de serviços ofertados, ainda há no Brasil alta proporção de serviços mutiladores em comparação a procedimentos que seriam alternativas às extrações dentárias ^31^. Apesar disso, nesta amostra, os procedimentos de exodontias foram os que tiveram menor percentual de aumento.

Os achados desse estudo mostram que políticas voltadas a grupos desfavorecidos podem não ter o efeito desejado e, por conseguinte, podem não reduzir desigualdades de uso e acesso. Assim, eles confirmam achados prévios que sugerem que o aumento de acesso à saúde bucal na APS pode não reduzir ou eliminar as desigualdades no uso dos serviços ^18^. Teoricamente, ofertar prioritariamente serviços às populações mais vulneráveis, incluído o grupo de beneficiários do Programa Bolsa Família, deveria reduzir desigualdades de acesso, se não houvesse barreiras específicas. O *Caderno da Saúde Bucal* do Ministério da Saúde sugere que as informações do Ministério do Desenvolvimento Social, à frente do Programa Bolsa Família na época, sejam utilizadas para organização do acesso local ^32^. É importante questionar se os serviços de saúde bucal estão realmente organizados de forma a atender os mais doentes ^33^. Ademais, o papel da intersetorialidade na gestão do programa ainda é deficiente, apresentando baixo envolvimento de outras áreas, como cultura e meio ambiente, e outros atores, como conselheiros e lideranças comunitárias, na promoção de oportunidades para o desenvolvimento dos beneficiários.

Uma limitação deste artigo é a possibilidade de subnotificação e erro de notificação nos sistemas consultados para obtenção dos dados de procedimentos, e tais erros tendem a reduzir a magnitude das associações. Assim, as associações relatadas podem estar subestimadas. Por outro lado, a dicotomização dos procedimentos odontológicos tende a minimizar erros de mensuração, introduzidos pela falta de padronização das notificações, removendo variabilidade não explicável por variáveis do modelo. Adicionalmente, o perfil do estudo em nível municipal não permite inferências em nível individual sem incorrer em falácia ecológica. Por fim, não há referencial teórico específico sobre os impactos de programas de transferência de renda, nem em saúde bucal, nem em uso de serviços ^11^. Dessa forma, a escolha de variáveis de ajuste foi realizada com base em potenciais fatores associados ao uso de serviços em nível individual. São pontos fortes deste estudo a inclusão de dados de todos os municípios brasileiros, excluindo a possibilidade de viés de seleção e perda de representatividade, e a utilização como exposição da medida de cobertura do Programa Bolsa Família, o qual atinge uma parcela da população que acessa os serviços de saúde odontológicos majoritariamente via SUS, que é a origem dos nossos dados de uso do serviço.

Ainda não se sabe quais componentes do programa impactam o uso de serviços, qual é a magnitude desses impactos, nem mesmo por quais vias eles ocorrem. Os efeitos já observados podem ser oriundos do aumento da renda e suas consequências ^34^ ou provocados pela influência das condicionalidades, que direcionam o usuário para um contato mais frequente com um serviço de saúde de referência. Novas pesquisas devem levar em conta, então, se o valor transferido é suficiente para impactar os desfechos avaliados, se o período de recebimento é suficiente para observar associações esperadas e se as condicionalidades podem ser um caminho entre o recebimento dos benefícios e o uso de serviços de saúde.
